# Effects of reactive oxygen species and mitochondrial dysfunction on reproductive aging

**DOI:** 10.3389/fcell.2024.1347286

**Published:** 2024-02-23

**Authors:** Jiangbo Song, Li Xiao, Zhehao Zhang, Yujin Wang, Panayiotis Kouis, Lene Juel Rasmussen, Fangyin Dai

**Affiliations:** ^1^ State Key Laboratory of Resource Insects, Key Laboratory of Sericultural Biology and Genetic Breeding, Ministry of Agriculture and Rural Affairs, College of Sericulture, Textile and Biomass Sciences, Southwest University, Chongqing, China; ^2^ Center for Healthy Aging, Department of Cellular and Molecular Medicine, University of Copenhagen, Copenhagen, Denmark

**Keywords:** aging, mitochondria, ROS, gamete quality, fertility, reproductive health

## Abstract

Mitochondria, the versatile organelles crucial for cellular and organismal viability, play a pivotal role in meeting the energy requirements of cells through the respiratory chain located in the inner mitochondrial membrane, concomitant with the generation of reactive oxygen species (ROS). A wealth of evidence derived from contemporary investigations on reproductive longevity strongly indicates that the aberrant elevation of ROS level constitutes a fundamental factor in hastening the aging process of reproductive systems which are responsible for transmission of DNA to future generations. Constant changes in redox status, with a pro-oxidant shift mainly through the mitochondrial generation of ROS, are linked to the modulation of physiological and pathological pathways in gametes and reproductive tissues. Furthermore, the quantity and quality of mitochondria essential to capacitation and fertilization are increasingly associated with reproductive aging. The article aims to provide current understanding of the contributions of ROS derived from mitochondrial respiration to the process of reproductive aging. Moreover, understanding the impact of mitochondrial dysfunction on both female and male fertility is conducive to finding therapeutic strategies to slow, prevent or reverse the process of gamete aging, and thereby increase reproductive longevity.

## 1 Introduction

Throughout history, aging has been a major concern for society. Healthcare advances and the distribution of vaccines have contributed to the increase in life expectancy from 35 years in the 18th century to 72.6 years today (Nakamura et al., 2017; Mansouri Torghabeh et al., 2022). Based on numerous observations, it has been hypothesized that the poor quality of reproductive cells can result in accelerated aging and a shorter healthspan (Loose et al., 2022). The state of the reproductive system is not only essential for fertility, but also for overall health. Reproductive aging is a universal developmental process conserved across species coinciding with age-related fertility depletion and decline of gamete quality, culminating in infertility and deleterious consequences on the offspring (Drori and Folman, 1976; Drewry et al., 2011; Archie et al., 2014; Sinha and Rae, 2014). Reproduction is an energy-costly undertaking that profoundly impacts on multiple individual characteristics at molecular, cellular, and endocrine levels (Loose et al., 2022; Secomandi et al., 2022). Nevertheless, reproductive fitness also offers notable physiological benefits. For instance, it is commonly discovered that women who remain reproductively healthy until their late 30 years or even 40 years have longer lifespans compared to younger women who give birth in their late 20 years (Murphy, 2023). Similarly, research on humans indicates that early menopause may be associated with increased mortality (Shuster et al., 2010). Despite the presence of numerous intrinsic and extrinsic factors that may contribute to aging traits in an organism’s germline, the intricate cellular mechanisms underlying reproductive aging remain poorly understood and warrant further investigation to fully comprehend the complex interactions involved.

In recent academic research, there is a growing emphasis on the role of mitochondria as a central component in cellular events associated with reproductive aging primarily due to oxidative stress (OS) caused by the progressive accumulation of ROS, as a result of oxidative phosphorylation (OxPhos) (Gumienny et al., 1999; Terman et al., 2007; Roger et al., 2017). Nevertheless, OS is not the sole contributing factor to mitochondria-dependent changes in reproductive aging; energy metabolism, mitochondrial dynamics, and the integrity of mitochondrial DNA (mtDNA) also play significant roles (Terman et al., 2007; Noh et al., 2023). Delayed blastocyst development and reduced quality in gametes are associated with accumulated oxidative damage and inefficient clearance of dysfunctional mitochondria (Shen et al., 2021; Khan et al., 2023). Importantly, there is a high probability that these issues are interconnected.

Here, we provide an overview of the evidence that explain how mitochondrial function and OS can affect reproductive function and the rate at which it undergoes cellular age-related alterations.

## 2 Mitochondria and ROS

### 2.1 The structure and functions of mitochondria

**FIGURE 1:**
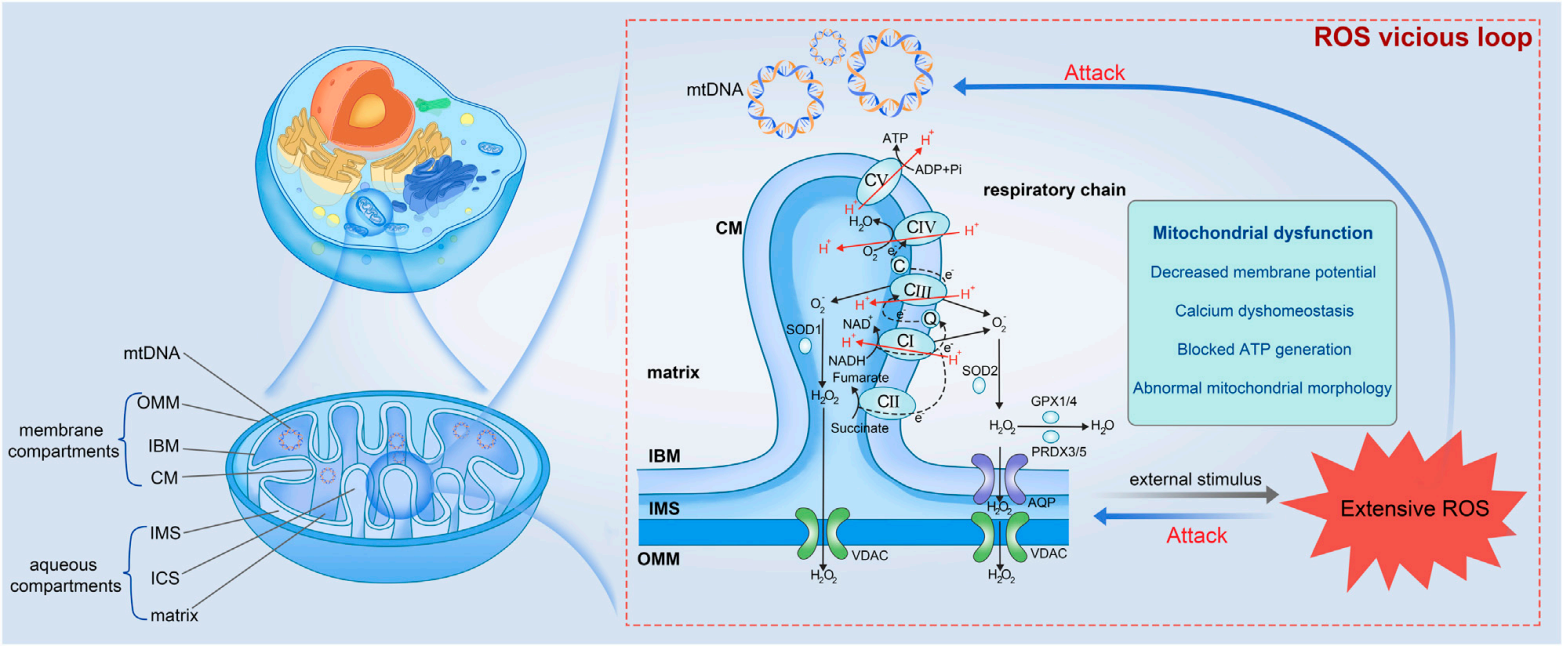
Mitochondrial ultrastructure and the vicious loop among extensive ROS, mito-structural damage, and mitochondrial dysfunction (partially refer to Iovine et al., 2021). Mitochondrion is precisely compartmentalized, which can be divided into mtDNA, membrane compartments and aqueous compartments. The membrane compartments include OMM, IBM, and CM from the outside in. The aqueous parts contain IMS, ICS, and the innermost matrix. Respiratory chain present within the IBM is composed of CI to V, which undergoes OxPhos for material and energy metabolism accompanied by ROS production. Once ROS is produced extensively and accumulated constantly in cells, ROS would damage the vulnerable mtDNA and mitochondrial inner structure, leading to mitochondrial dysfunction and more ROS. mtDNA, mitochondrial DNA; OMM, outer membrane; IBM, inner boundary membrane; CM, cristae membrane; IMS, intermembrane space; ICS, intracristal space; CI to V, Complex I to V; AQP, aquaporin; VDAC, voltage-dependent anion channel.

Mitochondria are ubiquitous intracellular organelles present in nearly all eukaryotes and involved in multiple aspects of cellular life, with a primary role in energy production. This functional diversity is reflected in the complicated mitochondrial ultrastructure (Figure 1). Mitochondria, unlike most organelles, are highlighted by their possession of two distinct membranes: an outer membrane (OMM) that encloses the organelle and interfaces with the cytosol, and an inner membrane (IMM) that displays morphological complexity. The IMM can be further subdivided into two components: the inner boundary membrane (IBM), which is closely attached to the outer membrane, and the cristae membrane (CM), which forms lamellar or tubular protrusion within the organelle. These membranes give rise to distinct compartments within the mitochondria, including the intermembrane space (IMS) located between the OMM and the IBM, the intracristal space (ICS) enclosed by the CM, and the innermost matrix (Iovine et al., 2021). Particularly, mitochondria possess their own DNA, which encodes numerous vital proteins necessary for the assembly and operation of mitochondrial respiratory complexes. MtDNA is especially vulnerable to stress-induced damage believed to be a result of the absence of histones in structure and limited effectiveness of repair mechanisms (Kim et al., 2015; Vadakedath et al., 2023).

Mitochondria are involved in essential functions of the cell, including energy metabolism, substance metabolism, signal transduction, intracellular calcium homeostasis, cell apoptosis (Iovine et al., 2021). Energy metabolism and material metabolism within mitochondria are synchronized. Mitochondria produce high-energy phosphorylated substance ATP into all cellular compartments through a complex interconnected metabolic network (TCA cycle, OxPhos, and fatty acid β-oxidation) using multiple energy sources, such as amino acids, lipids, and carbohydrate derivatives (Heldt, 1972; Wang J. et al., 2009). Mitochondria possess the ability to modulate energy production via glycolysis, the TCA cycle, and OxPhos in response to alterations in cellular energy demands (Lim et al., 2011). Furthermore, mitochondria assume a pivotal role in the regulation of intracellular signaling (Tan and Finkel, 2020). As a Ca^2+^ buffer zone within cells, mitochondria collaborate with the endoplasmic reticulum, extracellular matrix, and other cellular structures by Controlling the Ca^2+^ concentration to modulate cellular activities (Aoyama-Ishiwatari and Hirabayashi, 2021). Additionally, mitochondria participate in the regulation of various cellular stress responses, such as OS, heat stress, and apoptosis (Chien et al., 2014; Yao et al., 2023). The proper execution of mitochondrial functions counts on the structural compartmentalization, which allows mitochondria to respond to intracellular signals timely. Over the past decade, substantial evidence has emerged, shedding light on the involvement of mitochondria in gamete formation and embryonic development, as well as the elevated prevalence of reproductive disorders, such as inflammation of genitourinary system (Jankauskas et al., 2018), asthenospermia (Li et al., 2023), and polycystic ovary syndrome (Jiang et al., 2022).

### 2.2 Mitochondria as the major source of ROS generation

In eukaryotes, the production of ATP occurs within mitochondria through oxidative metabolism of nutrients, which involves two key steps: OxPhos process (Iovine et al., 2021), and the oxidation of nicotinamide adenine dinucleotide hydride (NADH) or flavin adenine nucleotide phosphate (FADH2), or the β-oxidation of fatty acids (Shen et al., 2017). The electron transport chain facilitates the oxidation of NADH and FADH2 generated through glycolysis and the TCA cycle. Simultaneously, it actively transports protons across the IMM, leading to the accumulation of protons in the IMS. This process establishes a transmembrane proton gradient with proton-motive force. Complex V (or called ATP synthase), harnesses the proton flow driven by this gradient to catalyze the synthesis of ATP from ADP and phosphoric acid. Consequently, the energy derived from the oxidation of hydrogen carriers is effectively conserved in the form of ATP (Figure 1).

From the Free radical theory of aging proposed by Harman early in 1956 focusing to the endogenous free radicals to the polished Mitochondrial theory of aging developed to include all forms of ROS today, intense research has approved that cumulative oxidative damage contributes to physiological deterioration and ultimately leads to aging and death, and mitochondria play an equally important role (Harman, 1956; Lee and Wei, 2001; Salmon et al., 2010; Holmstrom and Finkel, 2014). Concurrent alterations in ROS level and mitochondrial morphology have been reported in numerous experimental studies. For example, muscle- and skin-derived fibroblasts with an inactive Complex I exhibit an elevated ROS level and progressive mitochondrial fragmentation characterized by reduced mitochondrial length and branch, whereas patient cells without Complex I deficiency display only controllable ROS levels and a normal mitochondrial morphology (Koopman et al., 2007; Blanchet et al., 2011). The excessive presence of ROS heightens the likelihood of mutations of mtDNA that encodes proteins related to the respiratory chain, ultimately affecting ATP production and potent kinetic energy supply for motion (Venkatesh et al., 2009). Collectively, mitochondria as the primary generators and the recipients of oxidative damage, implies a vicious cycle wherein impaired mitochondria produce increased amounts of ROS, in turn leading to a progressive accumulation of damage that culminates in the process of aging (Figure 1).

In all cells, an intracellular balance between the production and elimination of ROS, known as “redox homeostasis”, necessitates efficient coordination of reactions across various cells and is regulated by an intricate network of pro-oxidation and antioxidant systems (Chianese and Pierantoni, 2021). The latter, the source of total antioxidant capacity, encompasses non-enzymatic scavengers, as well as detoxifying enzymes that operate within distinct organelles. Non-enzymatic antioxidants include albumin, urate, taurine, hypotaurine, pyruvate, lactate, tocopherol, ergothioneine, ascorbic acid and melatonin (Ball, 2008; Gonzalez-Arto et al., 2016). The antioxidant enzyme defense system involves superoxide dismutase (SOD), catalase (CAT), glutathione peroxidase (GPX), glutathione reductase (GSR), peroxiredoxin (PRDX), and so on (Ben Abdallah et al., 2009; Klopotowska et al., 2022). OS is defined as the imbalance between the detoxification of cell antioxidant defense system and the production of ROS. In the status of OS, extensive ROS is produced and accumulate within the whole cell. ROS is extremely reactive and can engage in reactions with virtually any substantial molecules, leading to lipid peroxidation and DNA impairment, among other consequences (Yang et al., 2010). Polyunsaturated fatty acid moieties of phospholipids and cholesterol are preferred substrates for lipid hydroperoxides (e.g., Malonaldehyde and 4-hydroxynonenal) and OH generated in membranes (Brouwers et al., 2005). Active ROS attacks directly sensitive DNA and damages components of the chromosome, such as telomeres, causing DNA strand breaks and altered repair mechanisms that can lead to the acquisition of genomic changes (Sallmyr et al., 2008). These impaired molecules disrupt regular cellular metabolism, prompting a subsequent escalation in ROS production, thus establishing a detrimental cycle. Ultimately, the excessive accumulation of ROS culminates in oxidative damage to macromolecules, cells, and even the overall structure and functionality of the organism (Redza-Dutordoir and Averill-Bates, 2016).

## 3 Effects of excessive ROS levels on reproductive aging

### 3.1 The role of ROS in oocyte quality and female fertility

**FIGURE 2:**
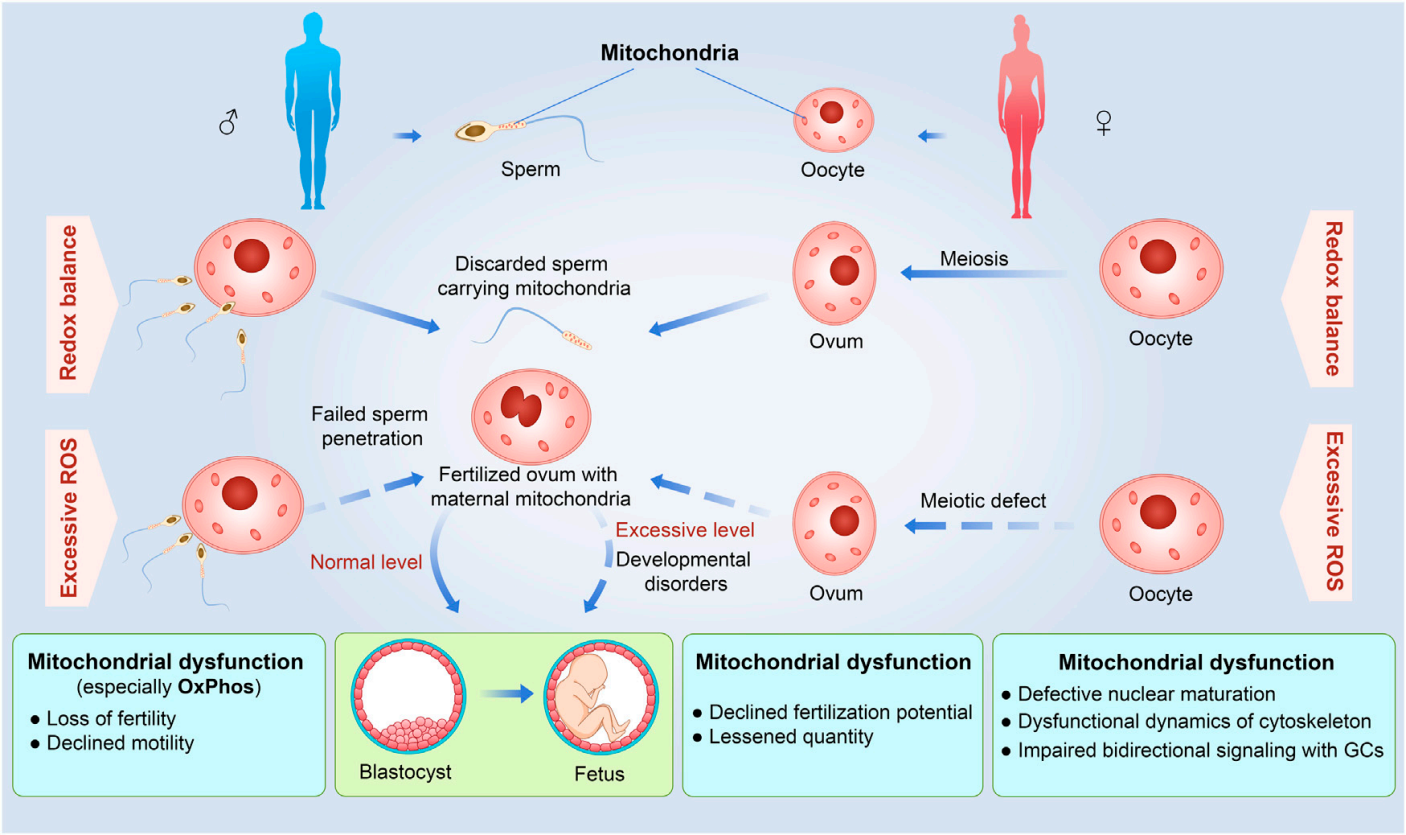
Detrimental effects of excessive ROS and mitochondrial dysfunction on different reproduction stages from gamete formation to embryonic development in the uterus. For the male, excessive ROS and mitochondrial dysfunction would cause inactive sperms with loss of fertility and declined motility by decreased energy supplement to fail to sperm penetration. For the female, excessive ROS and mitochondrial dysfunction would influence the entire oogenesis, increase the possibility of lessened quantity of ovum, and decline fertilization potential. Otherwise, excessive ROS can disturb the fetal development and even occurs developmental disorders. A solid arrow indicates that the process is normal, and a dashed arrow indicates that the process is defective.

ROS plays a dual role in oocyte development, serving as both a beneficial and detrimental factor. In its normal functioning, ROS assumes a crucial role in regulating folliculogenesis, meiosis, ovulation, and embryonic development by acting as secondary messengers in cellular signaling pathways (Agarwal et al., 2012). However, when present in excessive amounts, ROS will result in OS and exert detrimental effects including a series of reproductive diseases such as endometriosis and polycystic ovary syndrome (Wang F. et al., 2023; Weng et al., 2023; Zhou et al., 2023). The initial proposition by Tarin (Tarin, 1995) regarding ovarian senescence posits that oocytes exhibit reduced resistance to ROS, which is a significant contributor to oocyte impairment. The prolonged duration of meiosis-arrested oocytes increases their vulnerability to oxidative damage, as indicated by the disturbance of protein homeostasis, particularly in terms of proteolysis (Wu et al., 2022). Even though oocytes possess the capability to counteract this effect through the activation of intricate surveillance, repair, and proteolytic pathways, it is noteworthy that these defense systems are susceptible to age-related impairments, thereby diminishing their capacity to effectively eliminate proteins damaged by oxidation (Peters et al., 2020). ROS-induced damage can impact the primary molecular constituents of oocytes, encompassing lipid membranes, nucleic acids, and protein complexes, thereby disrupting protein functionality and cellular equilibrium (Husain and Mahmood, 2019; Cui et al., 2022). Increased ROS can also cause structural deterioration of organelles, leading to mitochondrial dysfunction, telomere shortening due to decreased telomerase activity, abnormalities in spindle assembly, and abnormal cytoskeletal dynamics (Zhang et al., 2018; Zhou et al., 2019). These abnormal conditions significantly diminish the effectiveness of meiotic recombination, resulting in errors in chromosome segregation and loss (Liu et al., 2004; Schatten and Sun, 2015; Yang et al., 2020). Consequently, oocytes undergo abnormal meiotic division and experience a substantial decline in egg quality (Figure 2).

The absence of antioxidant genes in the ovaries can disrupt follicular growth. Histological analysis conducted on the ovaries from SOD1 knockout mice demonstrated many primary and small antral follicles but few corpora lutea (Matzuk et al., 1998). Deletion of *SOD1* resulted in a defect in implantation of embryos to the wall of the uterine horns and small litters, suggesting that SOD1 may play a critical role in germ cell survival (Ho et al., 1998). The gilts supplied for dietary 2-hydroxy-4-methylselenobutanoic acid showed increased weight of uteri accompanied by increased the activity of T-AOC, T-SOD, GPX, TXNRD and decreased MDA content in granulosa cells (GCs), implied that the drug is conducive to promoting follicle development by antioxidant pathway (Lin et al., 2022). Furthermore, many advantageous factors from environment like resveratrol can elevate the level of ROS to generate various intracellular toxic effects including embryonic stage cellular toxicity and teratogenicity in various mammalian species and even the impact on human fetal development (Manzo et al., 2010; Auti et al., 2022). In recent years, more and more drugs have been evidenced playing a significantly antioxidant role to help delay or rescue the aging phenotypes of reproduction (Yang et al., 2022; Wang et al., 2023b; Wang et al., 2023c). For instance, the antioxidant Salidroside can increase the number of blastocyst cells, the proliferation of blastocysts, and the expressions of pluripotency genes while reducing ROS levels in oocytes and enhancing intracellular GSH levels (Shi et al., 2023). Currently, screening of drugs to effectively decrease the level of ROS possesses a broad prospect to ameliorate the quality of oocytes and their subsequent embryonic development.

### 3.2 The role of ROS in sperm functions and testis health

The decline in male reproductive function with age is a prevalent phenomenon observed in mammals, birds, and flies (Fricke and Koppik, 2019). Research conducted on males have revealed that aging poses smaller testes (Mahmoud et al., 2003), a notable decrease in the average quantity of sertoli cells (Johnson et al., 1984), and lower testosterone levels (Pines, 2011). A retrospective analysis of semen parameters (e.g., ejaculate volume, sperm concentration, and sperm motility), conducted on a sample size exceeding 5,000 men aged 16–72 years indicates semen parameters commence a decline after 34 years of age (Stone et al., 2013). Sperm quality, as one of the decisive factors affecting animal reproductive capacity, is crucial for the propagation across species. The abnormal increase of ROS in the reproductive system by age is one of the core causes of the decline of sperm quality.

Once ROS levels increase abnormally, it can lead to decreased fluidity of the mitochondrial membrane structure, protein denaturation, and increased membrane fragility within the sperm (Fleury et al., 2002). This disruption can directly result in the inability to maintain the proton gradient across the inner mitochondrial membrane, causing the biochemical basis of ATP synthesis to be compromised. As a result, sperm loses its motility and ability to move effectively due to decreased energy levels (Chianese and Pierantoni, 2021). In order to cope with the risk of high OS, sperm have developed a unique oxidative defense mechanism to maintain low levels of ROS within their internal environment (Guerriero et al., 2014; O’flaherty, 2019). Besides providing a liquid environment for sperms, seminal plasma serves as a source of numerous nutrients and protective substances that modulate sperm capacitation and gamete interaction (Vashisht and Gahlay, 2023). Among multitudinous substances, antioxidant system is high-profile for their roles involved in the process (Yin et al., 2023). In the seminal plasma of jackasses, the activities of antioxidant enzymes (SOD, CAT, GPX, and GSR) were significantly correlated with sperm motility and forward movement ability (Maehly and Chance, 1954; Wdowiak et al., 2015; Papas et al., 2019). The impairment by NiCl_2_ treatment on sperm total motility and progressive motility in a dose- and time-dependent manner is characterized by interference with Ca^2+^ signaling and elevated levels of ROS and malondialdehyde, which can be rescued by the antioxidants N-acetyl-L-cysteine or tocopherol (Chen et al., 2022). These studies indicate that antioxidant enzymes in the sperm storage organ play a strong protective role against OS ensuring maintenance of sperm vitality and overall quality. Understanding the regulatory mechanisms of the endogenous oxidative defense system, which serves as the primary barrier against high OS in sperm, is of significant value for improving reproductive capacity.

## 4 Mitochondrial dysfunctions associated with reproductive aging

### 4.1 Consequences of inadequate mtDNA levels

Mammalian mitochondria are naturally inherited through the maternal lineage (Hutchison et al., 1974), whereas the sperm mitochondria become degraded after fertilization (Sutovsky et al., 1999). Hence, the determination of oocyte health, fertilization, and early embryonic development hinges on the abundance and quality of the mitochondrial population within the oocyte to a great extent (Wai et al., 2010; Fragouli and Wells, 2015). The quality as well as the number of mitochondria within the oocyte play significant roles as markers for oocyte quality and pivotal indicators for fertilization and embryo development (Tilly and Sinclair, 2013). During the process of oocyte growth subsequent to follicle recruitment, the primordial oocyte experiences a substantial increase in the number of mitochondria (Wassarman et al., 1976). Hence, the replication of mtDNA and mitochondrial biogenesis is sustained in parallel with the follicle growth (Larsson et al., 1998; Hou et al., 2019). According to estimations, there is a decline of approximately 4 copies of human mtDNA every 10 years, which has been associated with compromised post-implantation development and an elevated likelihood of subfertility (Zhang et al., 2017; Busnelli et al., 2018). The analysis of an *in vitro* fertilization/intracytoplasmic sperm injection program suggested the circulating level of anti-Müllerian hormone, a hormone essential to the regulation of ovarian follicle growth by inhibiting initial follicle recruitment, is strongly correlated with mtDNA abundance within GCs (Bowolaksono et al., 2022). The accumulating evidence from current studies exhibits that an association exists between maternal age and the incidence of mtDNA deletion in oocytes and GCs (Keefe et al., 1995; Chan et al., 2005). Additionally, a greater number of mtDNA copies in the embryo is linked to higher fecundity (Murakoshi et al., 2013; Babayev et al., 2016). Generally, the inadequate amount of mtDNA is insufficient in supplying the required energy reserves for follicular growth, consequently resulting in oocyte degeneration.

### 4.2 Consequences of abnormal mitochondrial dynamics for the development of reproductive system

Mitochondria are organelles characterized by their dynamic nature with an intricate network of tubular structures, displaying an amazing plasticity of morphology and distribution. Mitochondrial dynamics includes the fusion and fission between the IBM and the OM during mitochondrial movement with surrounding mitochondria for exchange of matrix and membrane components (Wai and Langer, 2016). The two pivotal events maintain a continuously coordinated cycle essential for facilitating adequate local energy generation and distribution required by each cellular organelle to execute their respective functions (Frederick and Shaw, 2007). This enables mitochondria to preserve its optimal functioning status and structural integrity (Suen et al., 2008). Fusion serves to alleviate stress by amalgamating the constituents of partially impaired mitochondria, thereby enhancing their functionality, while fission is necessary for the elimination of compromised and less efficient mitochondria or for the generation of novel ones (Karbowski et al., 2002). Assessing the impact of mitochondrial fusion and fission on cellular processes proves challenging due to the multifaceted nature of these processes, which can yield diverse outcomes dependent on the temporal and spatial factors of their occurrence. It is worth noting that mitochondria are highly prevalent organelles in oocytes and embryos. Throughout maturation of oocytes and embryonic development preceding implantation, mitochondria undergo persistent and dynamic modifications to facilitate essential cellular developmental events (Sathananthan and Trounson, 2000; Harvey, 2019). The actional nature of energy demands leads to corresponding fluctuations in mitochondrial dynamics, which effectively and unitedly regulate metabolism to meet the material and energy requirements necessary for oocyte maturation and embryonic development (Schatten et al., 2005; Zhao et al., 2016).

The involvement of mitochondrial dynamic-related proteins in germ cells and embryos has been investigated extensively in mammals. The novel evidence from *mitofuscin 2* (*Mfn2*) knockout mice indicates that the seminiferous tubules display a vacuous morphology due to impaired spermatogenesis and increased apoptosis, parallel to an interference of cellular respiration effected by lower expression of the subunits of OxPhos complexes (Wang X. et al., 2021).

Indeed, it has been empirically shown that, alongside mitochondrial fusion, the process of fission is also necessary in governing the preservation of early spermatogenesis (Honda and Hirose, 2003). Stage-specific enhancement in *dynamin related protein 1* (*Drp1*) expression in rat spermatids cooperated with *Mfn2* to trigger the mitochondrial ubiquitination to attain their homogenization by fusion and fission. The *Drp1*-deleted oocytes displayed clustering of aberrant mitochondria, compromised calcium homeostasis, impaired secretory function, multiorganelle deformation, disrupted meiosis, and ultimately, age-dependent infertility in female mice (Udagawa et al., 2014). *Mitofuscin 1* (*Mfn1)* knockout oocytes exhibited impaired oocyte-GC communication, downregulated cadherins and connexins, resulting in follicle developmental arrest at the secondary follicle stage as well as depletion of ovarian follicular reserve. These phenotypes could be partially rescued by treatment with ceramide synthesis inhibitor myriocin (Zhang et al., 2019). Targeted deletion of *Mfn2* in mice showed reduced rates of oocyte maturation and fertilization, along with changes in mitochondrial allocation and spindle assembly (Liu et al., 2016), which highlighted the involvement of mitochondrial dynamics in regulating chromosome separation in oocytes. The activation of the embryonic genome may necessitate mitochondrial fusion to facilitate this significant developmental process. Inhibition of Drp1 in embryonic stem cells induced totipotency-to-pluripotency transition characterized by decreased activity of the glycolysis/gluconeogenesis pathway by failed silencing of 2-cell genes in embryos, ultimately leading to impeded development of early embryos (Guo et al., 2023). High expression of *Mfn1* significantly promoted early development of bovine embryos with somatic cell nuclear transfer by increasing ATP level and mitochondrial membrane potential, while reducing H_2_O_2_ generation by improving OxPhos (Babayev et al., 2016). Mitochondrial fission and fusion homeostasis are critical factors ensuring the optimal mitochondrial activity in oocytes and embryos, thereby facilitating the normal development of embryos, and preventing oxidative damage. With the identification and validation of additional molecules or drugs, the manipulation of mitochondrial dynamics may emerge as a prospective avenue for improving reproductive health.

### 4.3 Disruption of mitochondrial bioenergetics for oocyte maturation

It is well known that mitochondria are key regulators of structure formation, metabolic activity, and function (Sherratt, 1991). Their most primary role, energy production, is critical for function of organs with high metabolism like ovary and testis. The oocyte possesses a greater quantity of mitochondria compared to other cells in mammals (Kim et al., 2019). Research using mammalian models has provided compelling evidence that mitochondria are imperative for the development of proficient female gametes. During the maturation of follicles, as the efficiency of glycolysis in the oocyte is limited due to low expression of phosphofructokinase (Leese and Barton, 1984), there is a metabolic shift from glycolysis to OxPhos, which facilitates the production of increased ATP levels conducive to supporting various processes such as cytoplasmic and nuclear maturation, spindle formation, and polar body extrusion (Barbehenn et al., 1974; Zhang et al., 2006; Dumollard et al., 2007b). In this stage, there is a significant increase in the abundance of functional mitochondria, which serves as the primary provider of ATP for the reorganization of cytoplasmic and nuclear activities, such as gene expression and the initiation of crucial signaling cascades (Kirillova et al., 2021). Additionally, the spatial arrangement of mitochondria undergoes a transformation, transitioning from a homogenous distribution throughout the cytoplasm during the germinal vesicle stage to a clustered aggregation near the perinuclear region in metaphase I or II oocytes (Wang L. Y. et al., 2009).

Previously, it has been documented that there is a notable rise in ATP level alongside the clustering of mitochondria in the perinuclear region during the maturation of oocytes in mice and pigs (Yu et al., 2010). Inefficient mitochondrial bioenergetics can contribute to a detrimental impact on the effectiveness of bidirectional signal communications between GCs and oocytes, primarily mediated by the secreted sex hormones and intercellular gap junctions (Best et al., 2015). Consequently, this disruption may result in impairments in oocyte maturation. In addition, it has been demonstrated that a decline in ATP content generated through OxPhos process can impede the cytoplasmic microtubule network, leading to a delayed or hindered maturation of oocytes in mice (Marques-da-Silva et al., 2011). Disruption of OxPhos, the core process for ATP production, can cause halting of oocyte maturation, chromosomal misdistribution, and hindered embryo development (Takeuchi et al., 2005; Thouas et al., 2006; Wyman et al., 2008).The conditional disruption of the *decaprenyl diphosphate synthase subunit 2* (*Pdss2*) gene, leading to a deficiency of CoQ in oocytes, overviews numerous phenotypic alterations typical of mitochondrial dysfunction in oocytes associated with reproductive aging, and could be reversed by the administration of CoQ10 (Ben-Meir et al., 2015). During the matured phase of oocyte, mitochondria undergo relocation to varying regions to fulfill localized energy requirements. The occurrence of rapid increases in ATP demand and subsequent mitochondrial redistribution are closely linked to the development of the oocyte (Figure 2). An adequate number of functional mitochondria makes up a decisive factor in determining the quality of the oocyte.

## 5 Discussion

### 5.1 Variations in the rates of reproductive aging exist between males and females

In mammals, the process of reproduction can be categorized into distinct stages, namely, sex differentiation, sexual maturation, germ cell development encompassing spermatogenesis and oogenesis, mating, fertilization, pregnancy, and parturition. The lifespan expenses of sustaining reproductive capability, and of reproduction itself primarily focus on maintaining a stable developmental environment and ensuring an adequate energy supply, as any deviation or mistake at any stage can impede successful reproduction (Zhang and Hood, 2016). The completion of reproduction necessitates the collaborative efforts of both females and males, although females typically assume greater responsibilities, such as embryonic development, parturition, and lactation. Intriguingly, in the context of human reproduction, men exhibit a prolonged fertility span throughout their lives, consistently generating sperm, whereas women experience a comparatively limited window of fertility. The decline in female fertility commences around the age range of 30–35, manifesting in an augmented likelihood of miscarriage, diminished chances of conception, and an expedited progression of reproductive senescence, characterized by an increased probability of oocyte aneuploidy (Mogessie, 2020). The different reproductive aging patterns between sexes may be associated with the global phenomenon that women outlive men worldwide (Thornton, 2019). Sex differences in life expectancy also share a a prevalent trend in many species, including mammals (Hoffman et al., 2013), birds (Liker and Szekely, 2005), and insects (Bateman, 1948). Males may be sacrificing access to resources to longer lifespan expectancy in favor of sustaining an extended reproductive potential. One explanation for trade-offs between longevity and reproduction is that male sex hormones reduce the lifespan of men, proved by a longer lifespan in castrated men (Min et al., 2012). Asymmetric inheritance of mtDNA presents a novel perspective for explaining the sex difference in lifespan expectancy: the accumulation of mtDNA mutations may potentially make an adverse consequence for males if they are neutral or advantageous for females (Innocenti et al., 2011). These effects may manifest directly, such as through mitochondrial function (Anderson et al., 2022), or indirectly, such as retroegulation of nuclear genome by mtDNA (Hamalainen et al., 2019). However, the substantial variations in the reproductive cycle and the pace of reproductive senescence among males and females have yet to be adequately elucidated and warrant further investigation.

### 5.2 ROS and mitochondria interact in ovarian aging

The female reproductive longevity is predominantly influenced by the ovarian health. A shortened reproductive longevity in female with diminished fertility or potential infertility can be attributed to the accelerated ovarian aging. Ovarian aging is characterized by a decline in oocyte quality, as well as a decrease in the quantity of follicles and oocytes. The theory of ovarian aging, initially proposed by Tarin, posits that oocytes exhibit a reduced capacity to withstand ROS, a crucial factor contributing to oocyte impairment (Tarin, 1995). At a fundamental level, ROS functions as a secondary messenger in cellular signaling and assumes a crucial role in facilitating folliculogenesis, meiosis, and ovulation processes (Ellis et al., 1987). However, the detrimental effects of excessive ROS can adversely affect the fundamental molecular components of oocytes, leading to the disruption of protein functionality and cellular homeostasis (e.g., telomerase) (Sasaki et al., 2019). The two hit “telomere theory of reproductive aging” proposed that oocytes experience a greater accumulation of environmental and endogenous oxidative damage throughout the lifetime of female individuals, which could be mediated by telomeres (Robinson et al., 2023). Telomeres undergo elongation through two distinct mechanisms, telomerase and alternative lengthening of telomeres. Alternative lengthening of telomeres is infrequently observed except telomeropathy. Sperms harbor the longest telomeres in the body, which are continuously generated by a telomerase-active progenitor, spermatogonia, throughout the male’s lifespan. In contrast, the telomerase activity in the female germline is initially low and decreases following the initiation of meiotic arrest (Liu et al., 2007; Jeon et al., 2022). Otherwise, telomere attrition may still occur due to the presence of ROS (Kordowitzki, 2021). Therefore, oocytes frequently carry short telomeres trapped into a risky survival situation. Even though this theory has some rationality, ROS may interact with the health of oocytes in more aspects than just telomere length. The disruption caused by ROS will trigger endoplasmic reticulum stress, autophagy, and proteasome dysfunction, culminating in follicular atresia and a decline in both the quality and quantity of oocytes (Sasaki et al., 2019). Furthermore, mitochondrial quality determines the potential of oocyte fertility and the developing embryo (Dumollard et al., 2007a). Mitochondrial dysfunction can give rise to heightened ROS level and the accumulation of damaged age-related proteins (Seli et al., 2019). Excessive ROS levels can prompt intracellular OS and contribute to a decline in the quantity of primordial follicles and the mortality of pre-implantation embryos, consequently diminishing the reproductive lifespan of females (Sasaki et al., 2019). The interplay between ROS, mtDNA damage, and mitochondrial dysfunction establishes a vicious cycle (Figure 1), the occurrence of which can initiate a cascading effect-an increase in ROS levels and mtDNA mutagenesis, ultimately resulting in a decline in reproductive capacity.

### 5.3 The role of paternal mitochondria in offspring development

Although reproductive aging in males may not receive as much attention as in females, it similarly leads to infertility and has detrimental effects on offspring. The gradual decline in male reproductive function with age is a widespread phenomenon observed across species (Fricke and Koppik, 2019). Research in males has identified smaller testes, a notable reduction in the average number of Sertoli cells, and decreased testosterone levels as consequences of aging (Mahmoud et al., 2003). During the differentiation of mammalian sperms, a significant portion of the cytoplasm is eliminated; however, a subset of mitochondria persists within the sperms, assuming a tubular arrangement in proximity to the middle flagellum (Amaral et al., 2013). Furthermore, OS has been associated with male reproductive disorders (Lou et al., 2006; Mansouri Torghabeh et al., 2022; Xu et al., 2022). A harmonious redox state as well as normal mitochondria are crucial to support spermatogenesis, epididymal transport, and subsequent activities following ejaculation (motility, capacitation, and acrosome reaction) (Pelliccione et al., 2011). When the balance is disrupted, the resulting OS will adversely affect sperms in various aspects such as the genome, epigenome, lipidome, and proteome, thereby contributing to male infertility (Wang et al., 2003; Lim et al., 2019). In most instances, the inheritance of mitochondria occurs maternally, whereas paternal mitochondria are eliminated. Nevertheless, the precise mechanisms responsible for the selective paternal mitochondrial elimination, remain largely elusive. It is traditionally considered that sperm mitochondria and their mtDNA should be removed early in the embryo by selective mitophagy to protect the embryo and offspring from paternal mtDNA damage due to vulnerability of sperm mtDNA to oxidative damage (Marchetti et al., 2002). Paternal mitochondria elimination is carried out through the involvement of autophagy receptors in ubiquitin-dependent and ubiquitin-independent manners (Wei et al., 2017). For example, an allophagy receptor, ALLO-1, accumulates on the paternal organelles and leads to leads to local autophagosome formation depending on the ubiquitin modification after fertilization (Sato et al., 2018). FNDC-1, the *Caenorhabditis elegans* ortholog to human FUNDC1 (FUN14 domain containing1), is a ubiquitin-independent mitophagy receptor on the OMM involved in mitochondrial quality control under hypoxic conditions, and plays a crucial role in selective degradation of paternal mitochondria by the asymmetric high-expression on sperm mitochondria (Lim et al., 2019). Nevertheless, the role of sperm mitochondria in the fertilization and their final fate after fertilization remain subjects of ongoing debate. Recent investigations conducted in transgenic mice showed that sperm mitochondria are not subjected to elimination by the early embryonic degradation mechanisms, ubiquitin-proteasome, and autophagy. Instead, sperm mitochondria may persist uniformly in certain cells until the morula stage, suggesting paternal mtDNA could be transmitted to offspring (Luo et al., 2013). Indisputably, mitochondria play a crucial role in the processes of sperm fertility and fertilization, and any aberration within the mitochondria has the potential to cause impairment of sperm fertility and subsequent male infertility.

### 5.4 ROS and mitochondria are therapeutic targets for improvement of reproductive aging

The association between mitochondrial function and ROS is particularly pronounced in the context of reproductive aging. Currently, strategies aimed at mitigating oxidative damage and enhancing mitochondrial quality control have emerged as prominent therapeutic approaches for addressing reproductive aging and related disorders in clinical settings. The measurement of ROS levels in follicular fluid can serve as a biochemical marker for assessing follicular metabolic age (Wang et al., 2012), and male infertility (Wang et al., 2003). Some natural compounds with antioxidant properties that are isolated from medicinal plants such as resveratrol, have also been shown to protect against ovarian aging through multiple mechanisms, including counteracting cytotoxicity (Banu et al., 2016), preventing the loss of the GCs (Ozatik et al., 2020), improving renewal capacity of oogonial stem cells (Wu et al., 2019), and diminishing ovarian inflammation (Said et al., 2016). Mitochondrial membrane potential and mtDNA copy number have been identified as potential biomarkers for the selection of superior embryos and improved pregnancy (Wang J. et al., 2021) as well as for enhancing the accuracy of diagnosing male infertility (Karimian and Babaei, 2020). Experts in reproductive medicine have proposed that mitochondrial transplantation could serve as an innovative approach to rejuvenating oocyte quality and addressing age-related infertility or recurrent *in vitro* fertilization failures (Kristensen et al., 2017; Labarta et al., 2019). This technique has demonstrated positive outcomes in humans and various animal species. The transfer of autologous or heterologous mitochondria successfully enhanced oocyte quality and improved *in vitro* fertilization results in a bovine embryo model (Srirattana and St John, 2018). In aged mice, MII oocytes were fertilized with significantly increased blastocyst ratio after intracytoplasmic sperm injection combined with mitochondria from endometrial mesenchymal stem cells (Zhang et al., 2023). Nevertheless, it is important to acknowledge that this technique is limited in its ability to effectively enhance blastocyst quality (Labarta et al., 2019). Furthermore, the safety and ethical considerations surrounding the manipulation of mitochondria and blastocyst quality necessitate a more comprehensive understanding of their interaction. In essence, understanding the molecular processes that govern the balance of ROS and mitochondrial function is crucial for maintaining reproductive health. This knowledge is essential for identifying key targets to improve the quality of gametes. By doing so, we can delve into the causes of reproductive aging and disorders, paving the way for effective therapeutic strategies.

## 6 Future perspectives

The factors driving diverse aging phenotypes, especially concerning reproductive aging, are expected to be complex, involving changes across various physiological systems. Although we can differentiate between extrinsic and intrinsic factors, it is likely that the levels of ROS and mitochondrial quality associated with reproductive aging are influenced by both forces, suggesting a significant interplay between them. Delving into the intricate interplay among ROS, mitochondria, and germline function will yield valuable insights into underlying molecular mechanism of reproductive aging. Further exploration is also conducive to lay the groundwork to address fertility issues in humans by exploring potential strategies from the point of ROS level and mitochondria.

## Abbreviations

CAT, catalase; CM, cristae membrane; Drp1, dynamin related protein 1; FADH2, flavin adenine nucleotide phosphate; FUNDC1, FUN14 domain containing1; GC, granulosa cell; GPX, glutathione peroxidase; GSR, glutathione reductase; IBM, inner boundary membrane; ICS, intracristal space; IMM, inner membrane; IMS, intermembrane space; Mfn1/2, mitofuscin 1/2; mtDNA, mitochondrial DNA; NADH, nicotinamide adenine dinucleotide hydride; OMM, outer membrane; OS, oxidative stress; OxPhos, oxidative phosphorylation; PRDX, peroxiredoxin; ROS, reactive oxygen species; SOD, superoxide.

## References

[B1] Agarwal A. Aponte-Mellado A. Premkumar B. J. Shaman A. Gupta S. (2012). The effects of oxidative stress on female reproduction: a review. Reprod. Biol. Endocrinol. 10, 49. 10.1186/1477-7827-10-49 22748101 PMC3527168

[B2] Amaral A. Lourenco B. Marques M. Ramalho-Santos J. (2013). Mitochondria functionality and sperm quality. Reproduction 146, R163–R174. 10.1530/REP-13-0178 23901129

[B3] Anderson L. Camus M. F. Monteith K. M. Salminen T. S. Vale P. F. (2022). Variation in mitochondrial DNA affects locomotor activity and sleep in *Drosophila melanogaster* . Hered. (Edinb) 129, 225–232. 10.1038/s41437-022-00554-w PMC9519576 35764697

[B4] Aoyama-Ishiwatari S. Hirabayashi Y. (2021). Endoplasmic reticulum-mitochondria contact sites-emerging intracellular signaling hubs. Front. Cell. Dev. Biol. 9, 653828. 10.3389/fcell.2021.653828 34095118 PMC8172986

[B5] Archie E. A. Altmann J. Alberts S. C. (2014). Costs of reproduction in a long-lived female primate: injury risk and wound healing. Behav. Ecol. Sociobiol. 68, 1183–1193. 10.1007/s00265-014-1729-4 25045201 PMC4097819

[B6] Auti A. Alessio N. Ballini A. Dioguardi M. Cantore S. Scacco S. (2022). Protective effect of resveratrol against hypoxia-induced neural oxidative stress. J. Pers. Med. 12, 1202. 10.3390/jpm12081202 35893296 PMC9330416

[B7] Babayev E. Wang T. Szigeti-Buck K. Lowther K. Taylor H. S. Horvath T. (2016). Reproductive aging is associated with changes in oocyte mitochondrial dynamics, function, and mtDNA quantity. Maturitas 93, 121–130. 10.1016/j.maturitas.2016.06.015 27523387 PMC5064871

[B8] Ball B. A. (2008). Oxidative stress, osmotic stress and apoptosis: impacts on sperm function and preservation in the horse. Anim. Reprod. Sci. 107, 257–267. 10.1016/j.anireprosci.2008.04.014 18524506

[B9] Banu S. K. Stanley J. A. Sivakumar K. K. Arosh J. A. Burghardt R. C. (2016). Resveratrol protects the ovary against chromium-toxicity by enhancing endogenous antioxidant enzymes and inhibiting metabolic clearance of estradiol. Toxicol. Appl. Pharmacol. 303, 65–78. 10.1016/j.taap.2016.04.016 27129868 PMC5830085

[B10] Barbehenn E. K. Wales R. G. Lowry O. H. (1974). The explanation for the blockade of glycolysis in early mouse embryos. Proc. Natl. Acad. Sci. U. S. A. 71, 1056–1060. 10.1073/pnas.71.4.1056 4275392 PMC388161

[B11] Bateman A. J. (1948). Intra-sexual selection in Drosophila. Hered. (Edinb) 2, 349–368. 10.1038/hdy.1948.21 18103134

[B12] Ben Abdallah F. Dammak I. Mallek Z. Attia H. Hentati B. Ammar-Keskes L. (2009). Effects of date seed oil on testicular antioxidant enzymes and epididymal sperm characteristics in male mice. Andrologia 41, 229–234. 10.1111/j.1439-0272.2009.00923.x 19601934

[B13] Ben-Meir A. Burstein E. Borrego-Alvarez A. Chong J. Wong E. Yavorska T. (2015). Coenzyme Q10 restores oocyte mitochondrial function and fertility during reproductive aging. Aging Cell. 14, 887–895. 10.1111/acel.12368 26111777 PMC4568976

[B14] Best M. W. Wu J. Pauli S. A. Kane M. A. Pierzchalski K. Session D. R. (2015). A role for retinoids in human oocyte fertilization: regulation of connexin 43 by retinoic acid in cumulus granulosa cells. Mol. Hum. Reprod. 21, 527–534. 10.1093/molehr/gav017 25877907 PMC4447995

[B15] Blanchet L. Buydens M. C. Smeitink J. A. Willems P. H. Koopman W. J. (2011). Isolated mitochondrial complex I deficiency: explorative data analysis of patient cell parameters. Curr. Pharm. Des. 17, 4023–4033. 10.2174/138161211798764870 22188452

[B16] Bowolaksono A. Sundari A. M. Fauzi M. Maidarti M. Wiweko B. Mutia K. (2022). Anti-Mullerian hormone independently affect mtDNA copy number in human granulosa cells. J. Ovarian Res. 15, 111. 10.1186/s13048-022-01047-4 36224631 PMC9558397

[B17] Brouwers J. F. Silva P. F. Gadella B. M. (2005). New assays for detection and localization of endogenous lipid peroxidation products in living boar sperm after BTS dilution or after freeze-thawing. Theriogenology 63, 458–469. 10.1016/j.theriogenology.2004.09.046 15626411

[B18] Busnelli A. Lattuada D. Rossetti R. Paffoni A. Persani L. Fedele L. (2018). Mitochondrial DNA copy number in peripheral blood: a potential non-invasive biomarker for female subfertility. J. Assist. Reprod. Genet. 35, 1987–1994. 10.1007/s10815-018-1291-5 30120634 PMC6240551

[B19] Chan C. C. Liu V. W. Lau E. Y. Yeung W. S. Ng E. H. Ho P. C. (2005). Mitochondrial DNA content and 4977 bp deletion in unfertilized oocytes. Mol. Hum. Reprod. 11, 843–846. 10.1093/molehr/gah243 16421213

[B20] Chen C. Li B. Huang R. Dong S. Zhou Y. Song J. (2022). Involvement of Ca(2+) and ROS signals in nickel-impaired human sperm function. Ecotoxicol. Environ. Saf. 231, 113181. 10.1016/j.ecoenv.2022.113181 35026585

[B21] Chianese R. Pierantoni R. (2021). Mitochondrial reactive oxygen species (ROS) production alters sperm quality. Antioxidants (Basel) 10, 92. 10.3390/antiox10010092 33440836 PMC7827812

[B22] Chien C. Y. Chien C. T. Wang S. S. (2014). Progressive thermopreconditioning attenuates rat cardiac ischemia/reperfusion injury by mitochondria-mediated antioxidant and antiapoptotic mechanisms. J. Thorac. Cardiovasc Surg. 148, 705–713. 10.1016/j.jtcvs.2013.12.065 24507988

[B23] Cui X. Zhang Y. Lu Y. Xiang M. (2022). ROS and endoplasmic reticulum stress in pulmonary disease. Front. Pharmacol. 13, 879204. 10.3389/fphar.2022.879204 35559240 PMC9086276

[B24] Drewry M. D. Williams J. M. Hatle J. D. (2011). Life-extending dietary restriction and ovariectomy result in similar feeding rates but different physiologic responses in grasshoppers. Exp. Gerontol. 46, 781–786. 10.1016/j.exger.2011.06.003 21742024 PMC3166998

[B25] Drori D. Folman Y. (1976). Environmental effects on longevity in the male rat: exercise, mating, castration and restricted feeding. Exp. Gerontol. 11, 25–32. 10.1016/0531-5565(76)90007-3 1278267

[B26] Dumollard R. Duchen M. Carroll J. (2007a). The role of mitochondrial function in the oocyte and embryo. Curr. Top. Dev. Biol. 77, 21–49. 10.1016/S0070-2153(06)77002-8 17222699

[B27] Dumollard R. Ward Z. Carroll J. Duchen M. R. (2007b). Regulation of redox metabolism in the mouse oocyte and embryo. Development 134, 455–465. 10.1242/dev.02744 17185319

[B28] Ellis L. Morgan D. O. Jong S. M. Wang L. H. Roth R. A. Rutter W. J. (1987). Heterologous transmembrane signaling by a human insulin receptor-v-ros hybrid in Chinese hamster ovary cells. Proc. Natl. Acad. Sci. U. S. A. 84, 5101–5105. 10.1073/pnas.84.15.5101 3299376 PMC298801

[B29] Fleury C. Mignotte B. Vayssiere J. L. (2002). Mitochondrial reactive oxygen species in cell death signaling. Biochimie 84, 131–141. 10.1016/s0300-9084(02)01369-x 12022944

[B30] Fragouli E. Wells D. (2015). Mitochondrial DNA assessment to determine oocyte and embryo viability. Semin. Reprod. Med. 33, 401–409. 10.1055/s-0035-1567821 26565384

[B31] Frederick R. L. Shaw J. M. (2007). Moving mitochondria: establishing distribution of an essential organelle. Traffic 8, 1668–1675. 10.1111/j.1600-0854.2007.00644.x 17944806 PMC3739988

[B32] Fricke C. Koppik M. (2019). Male reproductive ageing: a tale of the whole ejaculate. Reproduction 158, R219–R229. 10.1530/REP-18-0579 31370008

[B33] Gonzalez-Arto M. Vicente-Carrillo A. Martinez-Pastor F. Fernandez-Alegre E. Roca J. Miro J. (2016). Melatonin receptors MT1 and MT2 are expressed in spermatozoa from several seasonal and nonseasonal breeder species. Theriogenology 86, 1958–1968. 10.1016/j.theriogenology.2016.06.016 27448693

[B34] Guerriero G. Trocchia S. Abdel-Gawad F. K. Ciarcia G. (2014). Roles of reactive oxygen species in the spermatogenesis regulation. Front. Endocrinol. (Lausanne) 5, 56. 10.3389/fendo.2014.00056 24795696 PMC4001055

[B35] Gumienny T. L. Lambie E. Hartwieg E. Horvitz H. R. Hengartner M. O. (1999). Genetic control of programmed cell death in the *Caenorhabditis elegans* hermaphrodite germline. Development 126, 1011–1022. 10.1242/dev.126.5.1011 9927601

[B36] Guo S. M. Zhang Y. R. Ma B. X. Zhou L. Q. Yin Y. (2023). Regulation of cleavage embryo genes upon DRP1 inhibition in mouse embryonic stem cells. Front. Cell. Dev. Biol. 11, 1191797. 10.3389/fcell.2023.1191797 37255603 PMC10225531

[B37] Hamalainen R. H. Landoni J. C. Ahlqvist K. J. Goffart S. Ryytty S. Rahman M. O. (2019). Defects in mtDNA replication challenge nuclear genome stability through nucleotide depletion and provide a unifying mechanism for mouse progerias. Nat. Metab. 1, 958–965. 10.1038/s42255-019-0120-1 32694840

[B38] Harman D. (1956). Aging: a theory based on free radical and radiation chemistry. J. Gerontol. 11, 298–300. 10.1093/geronj/11.3.298 13332224

[B39] Harvey A. J. (2019). Mitochondria in early development: linking the microenvironment, metabolism and the epigenome. Reproduction 157, R159–R179. 10.1530/REP-18-0431 30870807

[B40] Heldt H. W. (1972). Energy metabolism in mitochondria. Angew. Chem. Int. Ed. Engl. 11, 792–798. 10.1002/anie.197207921 4628612

[B41] Ho Y. S. Gargano M. Cao J. Bronson R. T. Heimler I. Hutz R. J. (1998). Reduced fertility in female mice lacking copper-zinc superoxide dismutase. J. Biol. Chem. 273, 7765–7769. 10.1074/jbc.273.13.7765 9516486

[B42] Hoffman J. M. Creevy K. E. Promislow D. E. (2013). Reproductive capability is associated with lifespan and cause of death in companion dogs. PLoS One 8, e61082. 10.1371/journal.pone.0061082 23613790 PMC3629191

[B43] Holmstrom K. M. Finkel T. (2014). Cellular mechanisms and physiological consequences of redox-dependent signalling. Nat. Rev. Mol. Cell. Biol. 15, 411–421. 10.1038/nrm3801 24854789

[B44] Honda S. Hirose S. (2003). Stage-specific enhanced expression of mitochondrial fusion and fission factors during spermatogenesis in rat testis. Biochem. Biophys. Res. Commun. 311, 424–432. 10.1016/j.bbrc.2003.10.008 14592431

[B45] Hou X. Zhu S. Zhang H. Li C. Qiu D. Ge J. (2019). Mitofusin1 in oocyte is essential for female fertility. Redox Biol. 21, 101110. 10.1016/j.redox.2019.101110 30690319 PMC6351231

[B46] Husain N. Mahmood R. (2019). Copper(II) generates ROS and RNS, impairs antioxidant system and damages membrane and DNA in human blood cells. Environ. Sci. Pollut. Res. Int. 26, 20654–20668. 10.1007/s11356-019-05345-1 31104239

[B47] Hutchison C. A. Newbold J. E. Potter S. S. Edgell M. H. (1974). Maternal inheritance of mammalian mitochondrial DNA. Nature 251, 536–538. 10.1038/251536a0 4423884

[B48] Innocenti P. Morrow E. H. Dowling D. K. (2011). Experimental evidence supports a sex-specific selective sieve in mitochondrial genome evolution. Science 332, 845–848. 10.1126/science.1201157 21566193

[B49] Iovine J. C. Claypool S. M. Alder N. N. (2021). Mitochondrial compartmentalization: emerging themes in structure and function. Trends Biochem. Sci. 46, 902–917. 10.1016/j.tibs.2021.06.003 34244035 PMC11008732

[B50] Jankauskas S. S. Silachev D. N. Andrianova N. V. Pevzner I. B. Zorova L. D. Popkov V. A. (2018). Aged kidney: can we protect it? Autophagy, mitochondria and mechanisms of ischemic preconditioning. Cell. Cycle 17, 1291–1309. 10.1080/15384101.2018.1482149 29963970 PMC6110592

[B51] Jeon H. J. Kang M. Kim J. S. Oh J. S. (2022). TCTP overexpression reverses age-associated telomere attrition by upregulating telomerase activity in mouse oocytes. J. Cell. Physiol. 237, 833–845. 10.1002/jcp.30557 34407217

[B52] Jiang X. L. Tai H. Xiao X. S. Zhang S. Y. Cui S. C. Qi S. B. (2022). Cangfudaotan decoction inhibits mitochondria-dependent apoptosis of granulosa cells in rats with polycystic ovarian syndrome. Front. Endocrinol. (Lausanne) 13, 962154. 10.3389/fendo.2022.962154 36465612 PMC9716878

[B53] Johnson L. Zane R. S. Petty C. S. Neaves W. B. (1984). Quantification of the human Sertoli cell population: its distribution, relation to germ cell numbers, and age-related decline. Biol. Reprod. 31, 785–795. 10.1095/biolreprod31.4.785 6509142

[B54] Karbowski M. Lee Y. J. Gaume B. Jeong S. Y. Frank S. Nechushtan A. (2002). Spatial and temporal association of Bax with mitochondrial fission sites, Drp1, and Mfn2 during apoptosis. J. Cell. Biol. 159, 931–938. 10.1083/jcb.200209124 12499352 PMC2173996

[B55] Karimian M. Babaei F. (2020). Large-scale mtDNA deletions as genetic biomarkers for susceptibility to male infertility: a systematic review and meta-analysis. Int. J. Biol. Macromol. 158, 85–93. 10.1016/j.ijbiomac.2020.04.216 32360203

[B56] Keefe D. L. Niven-Fairchild T. Powell S. Buradagunta S. (1995). Mitochondrial deoxyribonucleic acid deletions in oocytes and reproductive aging in women. Fertil. Steril. 64, 577–583. 10.1016/s0015-0282(16)57796-6 7641914

[B57] Khan S. A. Reed L. Schoolcraft W. B. Yuan Y. Krisher R. L. (2023). Control of mitochondrial integrity influences oocyte quality during reproductive aging. Mol. Hum. Reprod. 29, gaad028. 10.1093/molehr/gaad028 37594790

[B58] Kim H. R. Won S. J. Fabian C. Kang M. G. Szardenings M. Shin M. G. (2015). Mitochondrial DNA aberrations and pathophysiological implications in hematopoietic diseases, chronic inflammatory diseases, and cancers. Ann. Lab. Med. 35, 1–14. 10.3343/alm.2015.35.1.1 25553274 PMC4272938

[B59] Kim K. B. Dunn C. T. Park K. S. (2019). Recent progress in mapping the emerging landscape of the small-cell lung cancer genome. Exp. Mol. Med. 51, 1–13. 10.1038/s12276-019-0349-5 PMC6906379 31827074

[B60] Kirillova A. Smitz J. E. J. Sukhikh G. T. Mazunin I. (2021). The role of mitochondria in oocyte maturation. Cells 10, 2484. 10.3390/cells10092484 34572133 PMC8469615

[B61] Klopotowska M. Bajor M. Graczyk-Jarzynka A. Kraft A. Pilch Z. Zhylko A. (2022). PRDX-1 supports the survival and antitumor activity of primary and CAR-modified NK cells under oxidative stress. Cancer Immunol. Res. 10, 228–244. 10.1158/2326-6066.CIR-20-1023 34853030 PMC9414282

[B62] Koopman W. J. Verkaart S. Visch H. J. Van Emst-De Vries S. Nijtmans L. G. Smeitink J. A. (2007). Human NADH:ubiquinone oxidoreductase deficiency: radical changes in mitochondrial morphology? Am. J. Physiol. Cell. Physiol. 293, C22–C29. 10.1152/ajpcell.00194.2006 17428841

[B63] Kordowitzki P. (2021). Oxidative stress induces telomere dysfunction and shortening in human oocytes of advanced age donors. Cells, 10. 10.3390/cells10081866 34440635 PMC8391391

[B64] Kristensen S. G. Pors S. E. Andersen C. Y. (2017). Improving oocyte quality by transfer of autologous mitochondria from fully grown oocytes. Hum. Reprod. 32, 725–732. 10.1093/humrep/dex043 28333265

[B65] Labarta E. De Los Santos M. J. Escriba M. J. Pellicer A. Herraiz S. (2019). Mitochondria as a tool for oocyte rejuvenation. Fertil. Steril. 111, 219–226. 10.1016/j.fertnstert.2018.10.036 30611551

[B66] Larsson N. G. Wang J. Wilhelmsson H. Oldfors A. Rustin P. Lewandoski M. (1998). Mitochondrial transcription factor A is necessary for mtDNA maintenance and embryogenesis in mice. Nat. Genet. 18, 231–236. 10.1038/ng0398-231 9500544

[B67] Lee H. C. Wei Y. H. (2001). Mitochondrial alterations, cellular response to oxidative stress and defective degradation of proteins in aging. Biogerontology 2, 231–244. 10.1023/a:1013270512172 11868898

[B68] Leese H. J. Barton A. M. (1984). Pyruvate and glucose uptake by mouse ova and preimplantation embryos. J. Reprod. Fertil. 72, 9–13. 10.1530/jrf.0.0720009 6540809

[B69] Li J. Xu X. Liu J. Zhang S. Wang T. Liu Y. (2023). The immunity-related GTPase IRGC mediates interaction between lipid droplets and mitochondria to facilitate sperm motility. FEBS Lett. 597, 1595–1605. 10.1002/1873-3468.14640 37195149

[B70] Liker A. Szekely T. (2005). Mortality costs of sexual selection and parental care in natural populations of birds. Evolution 59, 890–897. 10.1554/04-560 15926698

[B71] Lim H. Y. Ho Q. S. Low J. Choolani M. Wong K. P. (2011). Respiratory competent mitochondria in human ovarian and peritoneal cancer. Mitochondrion 11, 437–443. 10.1016/j.mito.2010.12.015 21211574

[B72] Lim Y. Rubio-Pena K. Sobraske P. J. Molina P. A. Brookes P. S. Galy V. (2019). Fndc-1 contributes to paternal mitochondria elimination in *C. elegans* . Dev. Biol. 454, 15–20. 10.1016/j.ydbio.2019.06.016 31233739 PMC6717525

[B73] Lin H. Y. Yang Y. N. Chen Y. F. Huang T. Y. Crawford D. R. Chuang H. Y. (2022). 2,3,5,4'-Tetrahydroxystilbene-2-*O*-β-D-Glucoside improves female ovarian aging. Front. Cell. Dev. Biol. 10, 862045. 10.3389/fcell.2022.862045 36111333 PMC9469098

[B74] Liu L. Bailey S. M. Okuka M. Munoz P. Li C. Zhou L. (2007). Telomere lengthening early in development. Nat. Cell. Biol. 9, 1436–1441. 10.1038/ncb1664 17982445

[B75] Liu L. Franco S. Spyropoulos B. Moens P. B. Blasco M. A. Keefe D. L. (2004). Irregular telomeres impair meiotic synapsis and recombination in mice. Proc. Natl. Acad. Sci. U. S. A. 101, 6496–6501. 10.1073/pnas.0400755101 15084742 PMC404073

[B76] Liu Q. Kang L. Wang L. Zhang L. Xiang W. (2016). Mitofusin 2 regulates the oocytes development and quality by modulating meiosis and mitochondrial function. Sci. Rep. 6, 30561. 10.1038/srep30561 27469431 PMC4965743

[B77] Loose J. A. Amrit F. R. G. Patil T. Yanowitz J. L. Ghazi A. (2022). Meiotic dysfunction accelerates somatic aging in *Caenorhabditis elegans* . Aging Cell. 21, e13716. 10.1111/acel.13716 36176234 PMC9649607

[B78] Lou J. G. Dong J. Zheng Y. C. Zhang S. M. Xiao W. Q. Zhou J. F. (2006). Increased oxidative stress and damage in patients with chronic bacterial prostatitis. Biomed. Environ. Sci. 19, 481–486. 10.1111/j.1467-842X.2006.tb00794.x 17319275

[B79] Luo S. M. Schatten H. Sun Q. Y. (2013). Sperm mitochondria in reproduction: good or bad and where do they go? J. Genet. Genomics 40, 549–556. 10.1016/j.jgg.2013.08.004 24238608

[B80] Maehly A. C. Chance B. (1954). The assay of catalases and peroxidases. Methods Biochem. Anal. 1, 357–424. 10.1002/9780470110171.ch14 13193536

[B81] Mahmoud A. M. Goemaere S. El-Garem Y. Van Pottelbergh I. Comhaire F. H. Kaufman J. M. (2003). Testicular volume in relation to hormonal indices of gonadal function in community-dwelling elderly men. J. Clin. Endocrinol. Metab. 88, 179–184. 10.1210/jc.2002-020408 12519849

[B82] Mansouri Torghabeh F. Rostamzadeh P. Davoudi S. Keivan M. Shokri-Asl V. (2022). Effects of Rosmarinus officinalis on orchitis following spermatic cord torsion-detorsion in male mice with emphasis on anti-inflammatory and antioxidant properties. Andrologia 54, e14252. 10.1111/and.14252 34554588

[B83] Manzo S. Buono S. Cremisini C. (2010). Cadmium, lead and their mixtures with copper: *Paracentrotus lividus* embryotoxicity assessment, prediction, and offspring quality evaluation. Ecotoxicology 19, 1209–1223. 10.1007/s10646-010-0506-z 20552397

[B84] Marchetti C. Obert G. Deffosez A. Formstecher P. Marchetti P. (2002). Study of mitochondrial membrane potential, reactive oxygen species, DNA fragmentation and cell viability by flow cytometry in human sperm. Hum. Reprod. 17, 1257–1265. 10.1093/humrep/17.5.1257 11980749

[B85] Marques-Da-Silva C. Chaves M. M. Castro N. G. Coutinho-Silva R. Guimaraes M. Z. (2011). Colchicine inhibits cationic dye uptake induced by ATP in P2X2 and P2X7 receptor-expressing cells: implications for its therapeutic action. Br. J. Pharmacol. 163, 912–926. 10.1111/j.1476-5381.2011.01254.x 21306580 PMC3130939

[B86] Matzuk M. M. Dionne L. Guo Q. Kumar T. R. Lebovitz R. M. (1998). Ovarian function in superoxide dismutase 1 and 2 knockout mice. Endocrinology 139, 4008–4011. 10.1210/endo.139.9.6289 9724058

[B87] Min K. J. Lee C. K. Park H. N. (2012). The lifespan of Korean eunuchs. Curr. Biol. 22, R792–R793. 10.1016/j.cub.2012.06.036 23017989

[B88] Mogessie B. (2020). Advances and surprises in a decade of oocyte meiosis research. Essays Biochem. 64, 263–275. 10.1042/EBC20190068 32538429

[B89] Murakoshi Y. Sueoka K. Takahashi K. Sato S. Sakurai T. Tajima H. (2013). Embryo developmental capability and pregnancy outcome are related to the mitochondrial DNA copy number and ooplasmic volume. J. Assist. Reprod. Genet. 30, 1367–1375. 10.1007/s10815-013-0062-6 23897005 PMC3824848

[B90] Murphy C. T. (2023). Aging research: a field grows up. PLoS Biol. 21, e3002132. 10.1371/journal.pbio.3002132 37252935 PMC10228804

[B91] Nakamura J. Kamiya H. Haneda M. Inagaki N. Tanizawa Y. Araki E. (2017). Causes of death in Japanese patients with diabetes based on the results of a survey of 45,708 cases during 2001-2010: report of the Committee on Causes of Death in Diabetes Mellitus. J. Diabetes Investig. 8, 397–410. 10.1111/jdi.12645 PMC5415491 28349643

[B92] Noh S. E. Lee S. J. Lee T. G. Park K. S. Kim J. H. (2023). Inhibition of cellular senescence hallmarks by mitochondrial transplantation in senescence-induced ARPE-19 cells. Neurobiol. Aging 121, 157–165. 10.1016/j.neurobiolaging.2022.11.003 36442417

[B93] O’flaherty C. (2019). Orchestrating the antioxidant defenses in the epididymis. Andrology 7, 662–668. 10.1111/andr.12630 31044545

[B94] Ozatik F. Y. Ozatik O. Yigitaslan S. Kaygisiz B. Erol K. (2020). Do resveratrol and dehydroepiandrosterone increase diminished ovarian reserve? Eurasian J. Med. 52, 6–11. 10.5152/eurasianjmed.2019.19044 32158305 PMC7051231

[B95] Papas M. Arroyo L. Bassols A. Catalan J. Bonilla-Correal S. Gacem S. (2019). Activities of antioxidant seminal plasma enzymes (SOD, CAT, GPX and GSR) are higher in jackasses than in stallions and are correlated with sperm motility in jackasses. Theriogenology 140, 180–187. 10.1016/j.theriogenology.2019.08.032 31479834

[B96] Pelliccione F. Micillo A. Cordeschi G. D'angeli A. Necozione S. Gandini L. (2011). Altered ultrastructure of mitochondrial membranes is strongly associated with unexplained asthenozoospermia. Fertil. Steril. 95, 641–646. 10.1016/j.fertnstert.2010.07.1086 20840880

[B97] Peters A. E. Mihalas B. P. Bromfield E. G. Roman S. D. Nixon B. Sutherland J. M. (2020). Autophagy in female fertility: a role in oxidative stress and aging. Antioxid. Redox Signal 32, 550–568. 10.1089/ars.2019.7986 31892284

[B98] Pines A. (2011). Male menopause: is it a real clinical syndrome? Climacteric 14, 15–17. 10.3109/13697137.2010.507442 20670200

[B99] Redza-Dutordoir M. Averill-Bates D. A. (2016). Activation of apoptosis signalling pathways by reactive oxygen species. Biochim. Biophys. Acta 1863, 2977–2992. 10.1016/j.bbamcr.2016.09.012 27646922

[B100] Robinson L. G. Kalmbach K. Sumerfield O. Nomani W. Wang F. Liu L. (2023). Telomere dynamics and reproduction. Fertil. Steril. 121, 4–11. 10.1016/j.fertnstert.2023.11.012 37993053

[B101] Roger A. J. Munoz-Gomez S. A. Kamikawa R. (2017). The origin and diversification of mitochondria. Curr. Biol. 27, R1177–R1192. 10.1016/j.cub.2017.09.015 29112874

[B102] Said R. S. El-Demerdash E. Nada A. S. Kamal M. M. (2016). Resveratrol inhibits inflammatory signaling implicated in ionizing radiation-induced premature ovarian failure through antagonistic crosstalk between silencing information regulator 1 (SIRT1) and poly(ADP-ribose) polymerase 1 (PARP-1). Biochem. Pharmacol. 103, 140–150. 10.1016/j.bcp.2016.01.019 26827941

[B103] Sallmyr A. Fan J. Rassool F. V. (2008). Genomic instability in myeloid malignancies: increased reactive oxygen species (ROS), DNA double strand breaks (DSBs) and error-prone repair. Cancer Lett. 270, 1–9. 10.1016/j.canlet.2008.03.036 18467025

[B104] Salmon A. B. Richardson A. Perez V. I. (2010). Update on the oxidative stress theory of aging: does oxidative stress play a role in aging or healthy aging? Free Radic. Biol. Med. 48, 642–655. 10.1016/j.freeradbiomed.2009.12.015 20036736 PMC2819595

[B105] Sasaki H. Hamatani T. Kamijo S. Iwai M. Kobanawa M. Ogawa S. (2019). Impact of oxidative stress on age-associated decline in oocyte developmental competence. Front. Endocrinol. (Lausanne) 10, 811. 10.3389/fendo.2019.00811 31824426 PMC6882737

[B106] Sathananthan A. H. Trounson A. O. (2000). Mitochondrial morphology during preimplantational human embryogenesis. Hum. Reprod. 15 (Suppl. 2), 148–159. 10.1093/humrep/15.suppl_2.148 11041521

[B107] Sato M. Sato K. Tomura K. Kosako H. Sato K. (2018). The autophagy receptor ALLO-1 and the IKKE-1 kinase control clearance of paternal mitochondria in *Caenorhabditis elegans* . Nat. Cell. Biol. 20, 81–91. 10.1038/s41556-017-0008-9 29255173

[B108] Schatten H. Prather R. S. Sun Q. Y. (2005). The significance of mitochondria for embryo development in cloned farm animals. Mitochondrion 5, 303–321. 10.1016/j.mito.2005.05.003 16150655

[B109] Schatten H. Sun Q. Y. (2015). Centrosome and microtubule functions and dysfunctions in meiosis: implications for age-related infertility and developmental disorders. Reprod. Fertil. Dev. 27, 934–943. 10.1071/RD14493 25903261

[B110] Secomandi L. Borghesan M. Velarde M. Demaria M. (2022). The role of cellular senescence in female reproductive aging and the potential for senotherapeutic interventions. Hum. Reprod. Update 28, 172–189. 10.1093/humupd/dmab038 34918084 PMC8888999

[B111] Seli E. Wang T. Horvath T. L. (2019). Mitochondrial unfolded protein response: a stress response with implications for fertility and reproductive aging. Fertil. Steril. 111, 197–204. 10.1016/j.fertnstert.2018.11.048 30691623

[B112] Shen G. Zhou L. Liu W. Cui Y. Xie W. Chen H. (2017). Di(2-ethylhexyl)phthalate alters the synthesis and β-oxidation of fatty acids and hinders ATP supply in mouse testes via UPLC-Q-exactive orbitrap MS-based metabonomics study. J. Agric. Food Chem. 65, 5056–5063. 10.1021/acs.jafc.7b01015 28609104

[B113] Shen Q. Liu Y. Li H. Zhang L. (2021). Effect of mitophagy in oocytes and granulosa cells on oocyte quality†. Biol. Reprod. 104, 294–304. 10.1093/biolre/ioaa194 33079172

[B114] Sherratt H. S. (1991). Mitochondria: structure and function. Rev. Neurol. Paris. 147, 417–430. 1962047

[B115] Shi S. Geng Z. Yu X. Hu B. Liu L. Chi Z. (2023). Salidroside supplementation affects *in vitro* maturation and preimplantation embryonic development by promoting meiotic resumption. Genes. (Basel) 14, 1729. 10.3390/genes14091729 37761869 PMC10530922

[B116] Shuster L. T. Rhodes D. J. Gostout B. S. Grossardt B. R. Rocca W. A. (2010). Premature menopause or early menopause: long-term health consequences. Maturitas 65, 161–166. 10.1016/j.maturitas.2009.08.003 19733988 PMC2815011

[B117] Sinha A. Rae R. (2014). A functional genomic screen for evolutionarily conserved genes required for lifespan and immunity in germline-deficient *C. elegans* . PLoS One 9, e101970. 10.1371/journal.pone.0101970 25093668 PMC4122342

[B118] Srirattana K. St John J. C. (2018). Additional mitochondrial DNA influences the interactions between the nuclear and mitochondrial genomes in a bovine embryo model of nuclear transfer. Sci. Rep. 8, 7246. 10.1038/s41598-018-25516-3 29740154 PMC5940817

[B119] Stone B. A. Alex A. Werlin L. B. Marrs R. P. (2013). Age thresholds for changes in semen parameters in men. Fertil. Steril. 100, 952–958. 10.1016/j.fertnstert.2013.05.046 23809502

[B120] Suen D. F. Norris K. L. Youle R. J. (2008). Mitochondrial dynamics and apoptosis. Genes. Dev. 22, 1577–1590. 10.1101/gad.1658508 18559474 PMC2732420

[B121] Sutovsky P. Moreno R. D. Ramalho-Santos J. Dominko T. Simerly C. Schatten G. (1999). Ubiquitin tag for sperm mitochondria. Nature 402, 371–372. 10.1038/46466 10586873

[B122] Takeuchi T. Neri Q. V. Katagiri Y. Rosenwaks Z. Palermo G. D. (2005). Effect of treating induced mitochondrial damage on embryonic development and epigenesis. Biol. Reprod. 72, 584–592. 10.1095/biolreprod.104.032391 15525817

[B123] Tan J. X. Finkel T. (2020). Mitochondria as intracellular signaling platforms in health and disease. J. Cell. Biol. 219, e202002179. 10.1083/jcb.202002179 32320464 PMC7199861

[B124] Tarin J. J. (1995). Aetiology of age-associated aneuploidy: a mechanism based on the 'free radical theory of ageing. Hum. Reprod. 10, 1563–1565. 10.1093/humrep/10.6.1563 7593539

[B125] Terman A. Gustafsson B. Brunk U. T. (2007). Autophagy, organelles and ageing. J. Pathol. 211, 134–143. 10.1002/path.2094 17200947

[B126] Thornton J. (2019). WHO report shows that women outlive men worldwide. Bmj 365, l1631. 10.1136/bmj.l1631 30952650

[B127] Thouas G. A. Trounson A. O. Jones G. M. (2006). Developmental effects of sublethal mitochondrial injury in mouse oocytes. Biol. Reprod. 74, 969–977. 10.1095/biolreprod.105.048611 16452460

[B128] Tilly J. L. Sinclair D. A. (2013). Germline energetics, aging, and female infertility. Cell. Metab. 17, 838–850. 10.1016/j.cmet.2013.05.007 23747243 PMC3756096

[B129] Udagawa O. Ishihara T. Maeda M. Matsunaga Y. Tsukamoto S. Kawano N. (2014). Mitochondrial fission factor Drp1 maintains oocyte quality via dynamic rearrangement of multiple organelles. Curr. Biol. 24, 2451–2458. 10.1016/j.cub.2014.08.060 25264261

[B130] Vadakedath S. Kandi V. Ca J. Vijayan S. Achyut K. C. Uppuluri S. (2023). Mitochondrial deoxyribonucleic acid (mtDNA), maternal inheritance, and their role in the development of cancers: a scoping review. Cureus 15, e39812. 10.7759/cureus.39812 37397663 PMC10314188

[B131] Vashisht A. Gahlay G. K. (2023). Understanding seminal plasma in male infertility: emerging markers and their implications. Andrology. 10.1111/andr.13563 38018348

[B132] Venkatesh S. Deecaraman M. Kumar R. Shamsi M. B. Dada R. (2009). Role of reactive oxygen species in the pathogenesis of mitochondrial DNA (mtDNA) mutations in male infertility. Indian J. Med. Res. 129, 127–137. 19293438

[B133] Wai T. Ao A. Zhang X. Cyr D. Dufort D. Shoubridge E. A. (2010). The role of mitochondrial DNA copy number in mammalian fertility. Biol. Reprod. 83, 52–62. 10.1095/biolreprod.109.080887 20130269 PMC2888963

[B134] Wai T. Langer T. (2016). Mitochondrial dynamics and metabolic regulation. Trends Endocrinol. Metab. 27, 105–117. 10.1016/j.tem.2015.12.001 26754340

[B135] Wang F. Yin Y. Nie X. Zou Y. Tong X. Tong Y. (2023a). Protocatechuic acid alleviates polycystic ovary syndrome symptoms in mice by PI3K signaling in granulosa cells to relieve ROS pressure and apoptosis. Gynecol. Endocrinol. 39, 2228917. 10.1080/09513590.2023.2228917 37406659

[B136] Wang J. Bai L. Li J. Sun C. Zhao J. Cui C. (2009a). Proteomic analysis of mitochondria reveals a metabolic switch from fatty acid oxidation to glycolysis in the failing heart. Sci. China C Life Sci. 52, 1003–1010. 10.1007/s11427-009-0140-2 19937197

[B137] Wang J. Jia R. Gong H. Celi P. Zhuo Y. Ding X. (2021a). The effect of oxidative stress on the chicken ovary: involvement of microbiota and melatonin interventions. Antioxidants (Basel) 10, 1422. 10.3390/antiox10091422 34573054 PMC8472688

[B138] Wang L. Y. Wang D. H. Zou X. Y. Xu C. M. (2009b). Mitochondrial functions on oocytes and preimplantation embryos. J. Zhejiang Univ. Sci. B 10, 483–492. 10.1631/jzus.B0820379 19585665 PMC2704965

[B139] Wang X. Sharma R. K. Gupta A. George V. Thomas A. J. Falcone T. (2003). Alterations in mitochondria membrane potential and oxidative stress in infertile men: a prospective observational study. Fertil. Steril. 80 (2), 844–850. 10.1016/s0015-0282(03)00983-x 14505763

[B140] Wang X. Wen Y. Zhang J. Swanson G. Guo S. Cao C. (2021b). MFN2 interacts with nuage-associated proteins and is essential for male germ cell development by controlling mRNA fate during spermatogenesis. Development 148, dev196295. 10.1242/dev.196295 33674260

[B141] Wang Y. Chen X. Baker J. S. Davison G. W. Xu S. Zhou Y. (2023b). Astaxanthin promotes mitochondrial biogenesis and antioxidant capacity in chronic high-intensity interval training. Eur. J. Nutr. 62, 1453–1466. 10.1007/s00394-023-03083-2 36650315

[B142] Wang Y. Xu J. Fan Z. Zhou X. Wang Z. Zhang H. (2023c). Unlocking the antioxidant potential of white tea and osmanthus flower: a novel polyphenol liquid preparation and its impact on km mice and their offspring. Foods 12, 4041. 10.3390/foods12214041 37959160 PMC10650671

[B143] Wang Z. A. Huang J. Kalderon D. (2012). Drosophila follicle stem cells are regulated by proliferation and niche adhesion as well as mitochondria and ROS. Nat. Commun. 3, 769. 10.1038/ncomms1765 22473013 PMC3518414

[B144] Wassarman P. M. Albertini D. F. Josefowicz W. J. Letourneau G. E. (1976). Cytochalasin B-induced pseudo-cleavage of mouse oocytes *in vitro*: asymmetric localization of mitochondria and microvilli associated with a stage-specific response. J. Cell. Sci. 21, 523–535. 10.1242/jcs.21.3.523 184100

[B145] Wdowiak A. Brzozowski I. Bojar I. (2015). Superoxide dismutase and glutathione peroxidase activity in pregnancy complicated by diabetes. Ann. Agric. Environ. Med. 22, 297–300. 10.5604/12321966.1152083 26094527

[B146] Wei Y. Chiang W. C. Sumpter R. Mishra P. Levine B. (2017). Prohibitin 2 is an inner mitochondrial membrane mitophagy receptor. Cell. 168, 224–238. 10.1016/j.cell.2016.11.042 28017329 PMC5235968

[B147] Weng Y. Zhang Y. Wang D. Wang R. Xiang Z. Shen S. (2023). Exercise-induced irisin improves follicular dysfunction by inhibiting IRE1α-TXNIP/ROS-NLRP3 pathway in PCOS. J. Ovarian Res. 16, 151. 10.1186/s13048-023-01242-x 37525261 PMC10388501

[B148] Wu M. Ma L. Xue L. Ye W. Lu Z. Li X. (2019). Resveratrol alleviates chemotherapy-induced oogonial stem cell apoptosis and ovarian aging in mice. Aging (Albany NY) 11, 1030–1044. 10.18632/aging.101808 30779707 PMC6382418

[B149] Wu Y. W. Li S. Zheng W. Li Y. C. Chen L. Zhou Y. (2022). Dynamic mRNA degradome analyses indicate a role of histone H3K4 trimethylation in association with meiosis-coupled mRNA decay in oocyte aging. Nat. Commun. 13, 3191. 10.1038/s41467-022-30928-x 35680896 PMC9184541

[B150] Wyman A. Pinto A. B. Sheridan R. Moley K. H. (2008). One-cell zygote transfer from diabetic to nondiabetic mouse results in congenital malformations and growth retardation in offspring. Endocrinology 149, 466–469. 10.1210/en.2007-1273 18039778 PMC2219313

[B151] Xu W. Sun T. Wang J. Wang T. Wang S. Liu J. (2022). GPX4 alleviates diabetes mellitus-induced erectile dysfunction by inhibiting ferroptosis. Antioxidants (Basel) 11, 1896. 10.3390/antiox11101896 36290619 PMC9598206

[B152] Yang L. Tan G. Y. Fu Y. Q. Feng J. H. Zhang M. H. (2010). Effects of acute heat stress and subsequent stress removal on function of hepatic mitochondrial respiration, ROS production and lipid peroxidation in broiler chickens. Comp. Biochem. Physiol. C Toxicol. Pharmacol. 151, 204–208. 10.1016/j.cbpc.2009.10.010 19883793

[B153] Yang Q. Cong L. Wang Y. Luo X. Li H. Wang H. (2020). Increasing ovarian NAD(+) levels improve mitochondrial functions and reverse ovarian aging. Free Radic. Biol. Med. 156, 1–10. 10.1016/j.freeradbiomed.2020.05.003 32492457

[B154] Yang Q. Xi Q. Wang M. Long R. Hu J. Li Z. (2022). Rapamycin improves the quality and developmental competence of mice oocytes by promoting DNA damage repair during *in vitro* maturation. Reprod. Biol. Endocrinol. 20, 67. 10.1186/s12958-022-00943-0 35436937 PMC9014618

[B155] Yao X. Zhu J. Li L. Yang B. Chen B. Bao E. (2023). Hsp90 protected chicken primary myocardial cells from heat-stress injury by inhibiting oxidative stress and calcium overload in mitochondria. Biochem. Pharmacol. 209, 115434. 10.1016/j.bcp.2023.115434 36708886

[B156] Yin T. Yue X. Li Q. Zhou X. Dong R. Chen J. (2023). The association between the levels of oxidative stress indicators (MDA, SOD, and GSH) in seminal plasma and the risk of idiopathic oligo-asthenotera-tozoospermia: does Cu or Se level alter the association? Biol. Trace Elem. Res. 10.1007/s12011-023-03888-6 37803189

[B157] Yu Y. Dumollard R. Rossbach A. Lai F. A. Swann K. (2010). Redistribution of mitochondria leads to bursts of ATP production during spontaneous mouse oocyte maturation. J. Cell. Physiol. 224, 672–680. 10.1002/jcp.22171 20578238 PMC3149123

[B158] Zhang M. Bener M. B. Jiang Z. Wang T. Esencan E. Scott R. Iii (2019). Mitofusin 1 is required for female fertility and to maintain ovarian follicular reserve. Cell. Death Dis. 10, 560. 10.1038/s41419-019-1799-3 31332167 PMC6646343

[B159] Zhang Q. Hao J. X. Liu B. W. Ouyang Y. C. Guo J. N. Dong M. Z. (2023). Supplementation of mitochondria from endometrial mesenchymal stem cells improves oocyte quality in aged mice. Cell. Prolif. 56, e13372. 10.1111/cpr.13372 36480483 PMC9977672

[B160] Zhang R. Wang Y. Ye K. Picard M. Gu Z. (2017). Independent impacts of aging on mitochondrial DNA quantity and quality in humans. BMC Genomics 18, 890. 10.1186/s12864-017-4287-0 29157198 PMC5697406

[B161] Zhang X. Wu X. Q. Lu S. Guo Y. L. Ma X. (2006). Deficit of mitochondria-derived ATP during oxidative stress impairs mouse MII oocyte spindles. Cell. Res. 16, 841–850. 10.1038/sj.cr.7310095 16983401

[B162] Zhang Y. Hood W. R. (2016). Current versus future reproduction and longevity: a re-evaluation of predictions and mechanisms. J. Exp. Biol. 219, 3177–3189. 10.1242/jeb.132183 27802148 PMC5091378

[B163] Zhang Y. Wang C. Jin Y. Yang Q. Meng Q. Liu Q. (2018). Activating the PGC-1α/TERT pathway by catalpol ameliorates atherosclerosis via modulating ROS production, DNA damage, and telomere function: implications on mitochondria and telomere link. Oxid. Med. Cell. Longev. 2018, 2876350. 10.1155/2018/2876350 30046372 PMC6036816

[B164] Zhao H. C. Ding T. Ren Y. Li T. J. Li R. Fan Y. (2016). Role of Sirt3 in mitochondrial biogenesis and developmental competence of human *in vitro* matured oocytes. Hum. Reprod. 31, 607–622. 10.1093/humrep/dev345 26787646

[B165] Zhou C. Zhang X. Chen Y. Liu X. Sun Y. Xiong B. (2019). Glutathione alleviates the cadmium exposure-caused porcine oocyte meiotic defects via eliminating the excessive ROS. Environ. Pollut. 255, 113194. 10.1016/j.envpol.2019.113194 31520902

[B166] Zhou L. H. Zou H. Hao J. Y. Huang Y. Zhang J. N. Xu X. H. (2023). Metformin inhibits ovarian granular cell pyroptosis through the miR-670-3p/NOX2/ROS pathway. Aging (Albany NY) 15, 4429–4443. 10.18632/aging.204745 37244286 PMC10258021

